# Hydrogen Fertilization with Hydrogen Nanobubble Water Improves Yield and Quality of Cherry Tomatoes Compared to the Conventional Fertilizers

**DOI:** 10.3390/plants13030443

**Published:** 2024-02-02

**Authors:** Min Li, Guanjie Zhu, Ziyu Liu, Longna Li, Shu Wang, Yuhao Liu, Wei Lu, Yan Zeng, Xu Cheng, Wenbiao Shen

**Affiliations:** 1 College of Life Sciences, Laboratory Center of Life Sciences, Nanjing Agricultural University, Nanjing 210095, China; 2021816130@stu.njau.edu.cn (M.L.); 2023816117@stu.njau.edu.cn (G.Z.); 2022116085@stu.njau.edu.cn (Z.L.); lln2013034@njau.edu.cn (L.L.); 2019816129@njau.edu.cn (S.W.); 2019816130@njau.edu.cn (Y.L.); luw@njau.edu.cn (W.L.); 2 Life Science Group, Air Liquide (China) R&D Co., Ltd., Shanghai 201108, China; yan.zeng@airliquide.com (Y.Z.); stven.cheng@airliquide.com (X.C.)

**Keywords:** cherry tomato, fertilizer, hydrogen, hydrogen nanobubble water, quality, yield

## Abstract

Although hydrogen gas (H_2_)-treated soil improves crop biomass, this approach appears difficult for field application due to the flammability of H_2_ gas. In this report, we investigated whether and how H_2_ applied in hydrogen nanobubble water (HNW) improves the yield and quality of cherry tomato (*Lycopersicon esculentum* var. *cerasiforme*) with and without fertilizers. Two-year-long field trials showed that compared to corresponding controls, HNW without and with fertilizers improved the cherry tomato yield per plant by 39.7% and 26.5% in 2021 (Shanghai), respectively, and by 39.4% and 28.2% in 2023 (Nanjing), respectively. Compared to surface water (SW), HNW increased the soil available nitrogen (N), phosphorus (P), and potassium (K) consumption regardless of fertilizer application, which may be attributed to the increased NPK transport-related genes in roots (*LeAMT2*, *LePT2*, *LePT5*, and *SlHKT1,1*). Furthermore, HNW-irrigated cherry tomatoes displayed a higher sugar–acid ratio (8.6%) and lycopene content (22.3%) than SW-irrigated plants without fertilizers. Importantly, the beneficial effects of HNW without fertilizers on the yield per plant (9.1%), sugar–acid ratio (31.1%), and volatiles (20.0%) and lycopene contents (54.3%) were stronger than those achieved using fertilizers alone. In short, this study clearly indicated that HNW-supplied H_2_ not only exhibited a fertilization effect on enhancing the tomato yield, but also improved the fruit’s quality with a lower carbon footprint.

## 1. Introduction

Cherry tomato (*Lycopersicon esculentum* var. *cerasiforme*), a small-fruited variety of tomato, is a popular and widely cultivated fruit vegetable in the world [1]. Since cherry tomato is rich in nutrients such as lycopene, vitamins, and minerals, it is favored by consumers for reducing the risk of various diseases, such as cardiovascular disorders, hypercholesterolemic and hyperglycemic attributes, and cancer [2]. Although fertilizers can improve the fruit yield, over-fertilization not only causes water pollution, but also results in flavor loss [3] and fruit nitrate and nitrite accumulation [4]. In addition, the large-scale application of fertilizers for crop production increases greenhouse gas emissions and accelerates global warming [5]. Therefore, it is a modern challenge to improve the tomato yield and quality in a more fertilizer-efficient and environmentally friendly way.

In the last decade, molecular hydrogen (H_2_) has been considered as a promising medical treatment for metabolic, digestive, respiratory, and cardiovascular diseases, neurodegenerative disorders, and cancer [6]. In addition, H_2_ exhibits a variety of biological functions in plants, including alleviating the oxidative damage caused by various abiotic stresses [7], promoting seed germination and root development [8], and improving the postharvest preservation of vegetables [9], fruits [10], and flowers [11]. It has been previously found that H_2_-exposed soils can promote the biomass of soybean, spring wheat, barley, and canola, suggesting that H_2_ has an effect of fertilizer utilization in soils [12,13]. However, H_2_ applied in a gaseous form for soil treatment is complicated and impractical in the field due to its low residency and flammable properties at higher concentrations.

Although the application of hydrogen-rich water (HRW) has been found to improve the yield and prolong the shelf life of daylily buds [14], it has the disadvantages of the low solubility and short residence time of dissolved H_2_. Solid H_2_ storage materials, such as magnesium hydride (MgH_2_) [15], ammonia borane (AB) [16], and AB-loaded hollow mesoporous silica nanoparticles (AB@hMSNs) [17], can improve the effective H_2_ residency of conventional HRW, and thus have positive effects on flower senescence, stress responses, and plant growth regulation. Nevertheless, the potential environmental risk of their by-products should be considered, especially when they are extensively used in the field.

The nanobubble technology establishes a useful approach to accelerate gas dissolution and remain its stability in the liquids for longer times [18]. Hydrogen nanobubble water (HNW) has been reported to reduce the toxicity of copper to *Daphnia magna* by alleviating oxidative stress and inhibiting copper accumulation [19]. Moreover, HNW can promote seed germination and concentrations of bioactive phytochemicals in sprouted black barley [20]. A solution of HNW was also shown to extend the vase life of cut carnation flowers [11]. A previous field trial showed that HNW increased the size and quality of rice grains [21], and enhanced the aroma of strawberries [22].

In this study, two-year and multi-site trials were carried out to investigate whether and how a preharvest HNW treatment improved the cherry tomato yield and quality (including sugars, vitamin C, lycopene, phenols, and flavonoids contents), in the absence (especially) or presence of fertilizers. The changes in available nitrogen (N), phosphorus (P), and potassium (K) in the soil, and transcriptional profiles of genes associated with tomato nutrition absorption and quality, were further investigated. The results thus provide a reference for the practical application of HNW in horticulture for better performance in terms of both yield and quality, which might open a new window for the low carbon society.

## 2. Results

### 2.1. Preharvest Application of HNW Improves Cherry Tomato Yield

**Figure 1:**
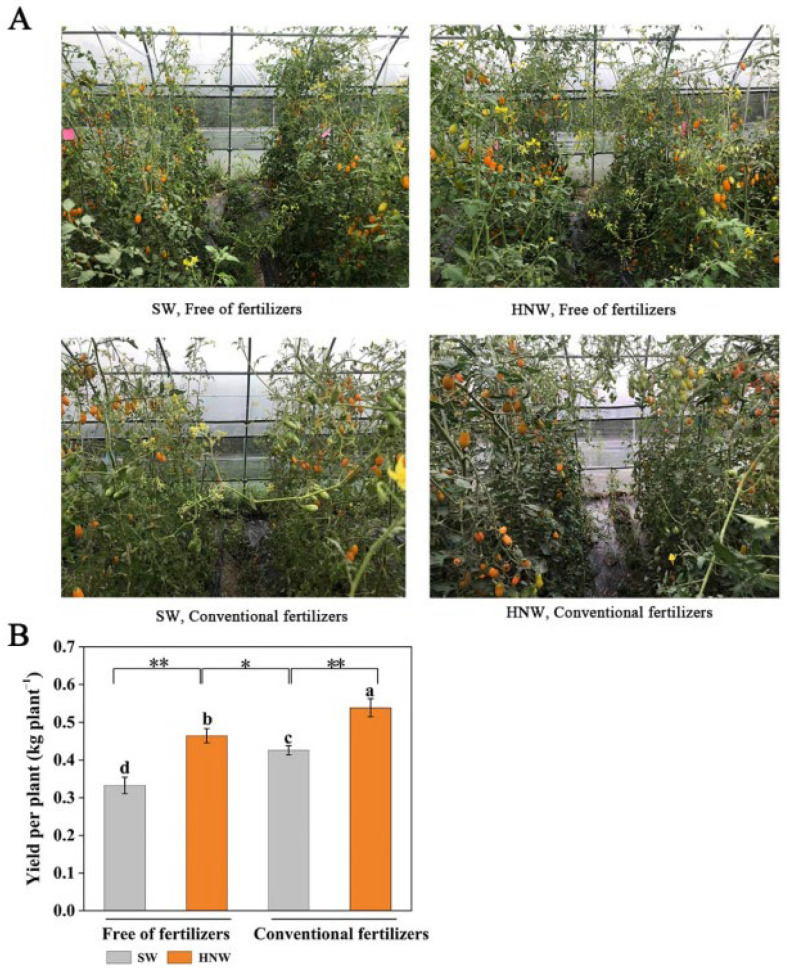
Hydrogen nanobubble water (HNW)—promoted cherry tomato growth (**A**) and yield per plant (**B**) with/without fertilizers (Shanghai, 2021). Values are mean ± SD of three independent experiments. The asterisks *, ** indicate significant differences at *p* < 0.05 and *p* < 0.01, respectively (*t*-test). The different letters indicate significant differences at *p* < 0.05 (one-way ANOVA; Duncan’s multiple range tests). SW: surface water; HNW: hydrogen nanobubble water.

As shown in Figure 1A, the irrigation with HNW promoted the growth of tomato plants in Shanghai (2021). Similar with compound fertilizers, HNW increased the yield per plant of cherry tomato (Figure 1B). Compared to SW irrigation, the yield per plant of cherry tomatoes in the treatment of HNW without fertilizers was increased by 39.7% (*p* < 0.01). Meanwhile, HNW plus fertilizers treatment showed the obvious effect on cherry tomato yield, and increased yield per plant by 26.5% in comparison with SW plus fertilizers (*p* < 0.01). The yield per plant in the treatment of HNW without fertilizers was even higher (9.1%, *p* < 0.05) than that of the SW plus fertilizers treatment.

**Figure 2:**
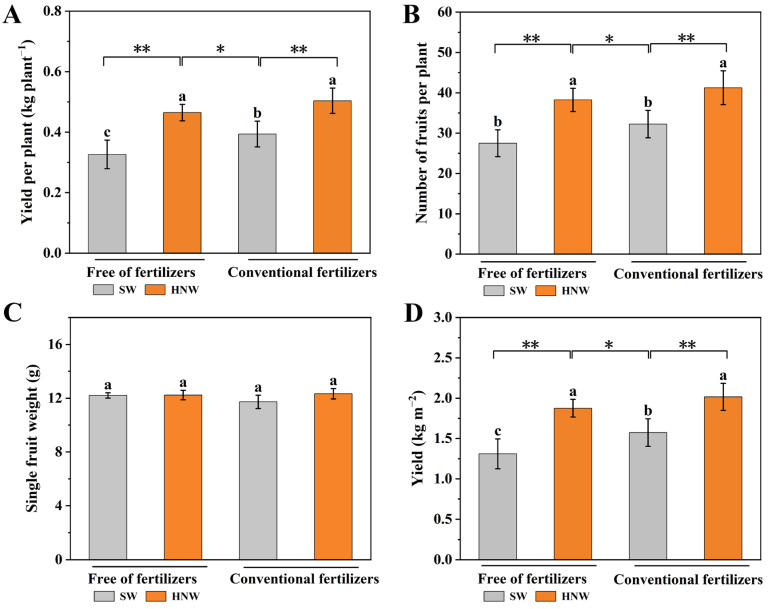
Effects of hydrogen nanobubble water on the yield per plant (**A**), number of fruits per plant (**B**), single fruit weight (**C**), and yield (**D**) of cherry tomatoes with/without fertilizers (Nanjing, 2023). Values are mean ± SD of three independent experiments. The asterisks *, ** indicate significant differences at *p* < 0.05 and *p* < 0.01, respectively (*t*-test). The different letters indicate significant differences at *p* < 0.05 (one-way ANOVA; Duncan’s multiple range tests). SW: surface water; HNW: hydrogen nanobubble water.

To further assess the reliability of the yield enhancement achieved using hydrogen-based irrigation in cherry tomato, we conducted another field trial test in Nanjing (2023). Consistently, the increase in yield per plant exhibited a similar trend as observed in the previous results (Figure 1). As shown in Figure 2A, under the conditions with and without fertilizers, HNW (0.50 ± 0.04 kg plant^−1^/0.46 ± 0.03 kg plant^−1^) remarkably resulted in the increase in the yield per plant by 28.2% (*p* < 0.01) and 39.4% (*p* < 0.01), respectively, compared with SW (0.39 ± 0.04 kg plant^−1^/0.33 ± 0.05 kg plant^−1^) in that year. In addition, the number of fruits per plant in the HNW treatment was also higher than in the SW treatment regardless of fertilizer use (Figure 2B). For example, compared with the SW alone and SW plus fertilizers group, HNW remarkably increased the number of fruits per plant by 39.10% (*p* < 0.01) and 27.91% (*p* < 0.01), respectively. Although HNW did not obviously influence the single fruit’s weight (Figure 2C), the increased numbers of fruits per plant might ultimately increase the cherry tomato yield (Figure 2D) by 43.3% and 28.1% in comparison with SW in the absence or presence of fertilizers, respectively. Comparatively, we clearly observed that the yield was higher in the HNW-irrigated and no fertilizer addition group than the SW-irrigated and fertilizer-added group (22.1%, *p* < 0.05), which could be partially explained by the obvious increases in yield per plant and number of fruits per plant compared to the fertilizers group (*p* < 0.05).

### 2.2. Effects of HNW on the Balance of Sugars and Acids in Cherry Tomatoes

**Figure 3:**
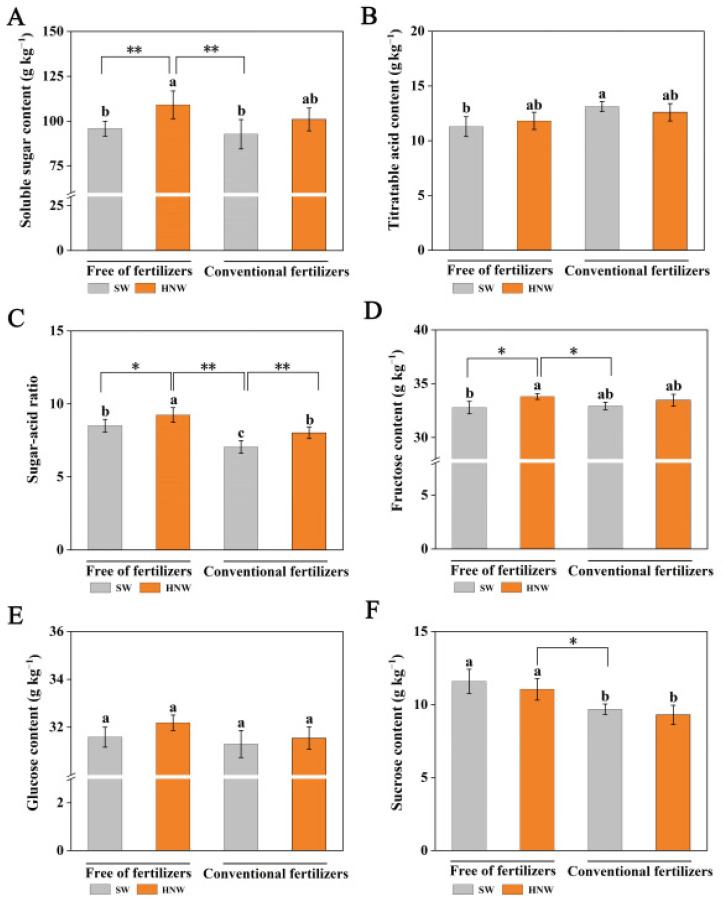
Effects of hydrogen nanobubble water on the contents of soluble sugar (**A**), titratable acid (**B**), sugar–acid ratio (**C**), fructose (**D**), glucose (**E**), and sucrose (**F**) in cherry tomatoes with/without fertilizers (Shanghai, 2021). Values are mean ± SD of three independent experiments. The asterisks *, ** indicate significant differences at *p* < 0.05 and *p* < 0.01, respectively (*t*-test). The different letters indicate significant differences at *p* < 0.05 (one-way ANOVA; Duncan’s multiple range tests). SW: surface water; HNW: hydrogen nanobubble water.

The main factors affecting the flavor of cherry tomatoes are the content and ratio of sugars and acids, which are critical to their commercial value [23]. In the absence of fertilizers, HNW treatment significantly increased the soluble sugar content (13.9%, *p* < 0.01; Figure 3A), and slightly increased the titratable acid content (4.7%; Figure 3B), thus causing the sugar–acid ratio to increase (8.6%, *p* < 0.05; Figure 3C). Comparatively, fertilizer addition alone had no such effect on the soluble sugar content, but increased the titratable acid content, resulting in a decreased sugar–acid ratio (−17.1%, *p* < 0.05; Figure 3C).

When HNW was applied without fertilizers, it clearly also showed the most obvious effect on increasing the sugar–acid ratio, especially with an increase of 31.1% in comparison with fertilizers alone (*p* < 0.01). Meanwhile, HNW impaired the negative effect of fertilizers on the sugar–acid ratio when it was applied in combination with fertilizers.

Fructose, glucose, and sucrose are the three main soluble sugars in cherry tomatoes [24]. Compared with SW alone, the HNW addition significantly increased the fructose content (3.1%, *p* < 0.05), but not that of glucose and sucrose (Figure 3D–F). Fertilizer application decreased the sucrose content (−16.4%, *p* < 0.05), while it did not influence the other two sugars. Therefore, the treatment of HNW without fertilizers had better effects on the fructose and sucrose content compared with fertilizers alone (*p* < 0.05). In the presence of fertilizers, HNW only slightly increased the fructose content. The observations above indicate that HNW might regulate the balance of sugars and acids in cherry tomato fruits.

### 2.3. Antioxidant Compounds Accumulation in Response to HNW

**Figure 4:**
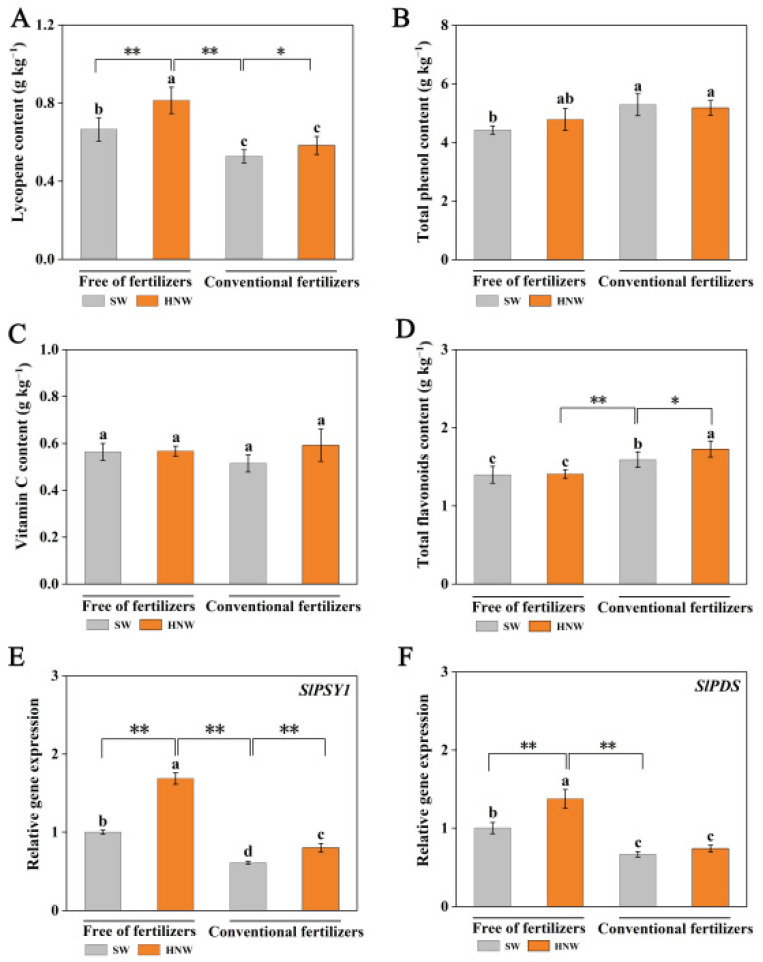
Effects of HNW on the accumulation of lycopene (**A**), total phenols (**B**), vitamin C (**C**), and flavonoids (**D**); and the expression level of *SlPSY1* (**E**) and *SlPDS* (**F**) in cherry tomatoes with/without fertilizers (Shanghai, 2021). Values are mean ± SD of three independent experiments. The asterisks *, ** indicate significant differences at *p* < 0.05 and *p* < 0.01, respectively (*t*-test). The different letters indicate significant differences at *p* < 0.05 (one-way ANOVA; Duncan’s multiple range tests). SW: surface water; HNW: hydrogen nanobubble water.

Cherry tomatoes contain numerous antioxidant ingredients such as ascorbic acid (vitamin C), phenols, and flavonoids, thus having antioxidative, anti-inflammatory, and anti-microbial effects [25]. Among them, lycopene has an outstanding contribution [26]. Further results showed that the application of HNW without fertilizers differentially increased the accumulation of lycopene (22.3%, *p* < 0.01; Figure 4A) and total phenols (8.1%; Figure 4B), except for vitamin C (Figure 4C) and flavonoids (Figure 4D). By contrast, under our experimental conditions, the application with fertilizers significantly decreased the lycopene content, and obviously increased the total phenols and flavonoids contents. Accordingly, in the absence of fertilizers, HNW significantly increased the lycopene content (54.3%, *p* < 0.01), but decreased the total flavonoid content (*p* < 0.01) in comparison with fertilizer alone. Meanwhile, when HNW was combined with fertilizers, only the flavonoids content was significantly increased.

Phytoene synthase (PSY) and phytoene desaturase (PDS) are the key enzymes in determining the biosynthesis of lycopene [27]. The changes in expression levels of *SlPSY1* and *SlPDS* displayed approximately similar trends compared to the lycopene content after the application of HNW with/without fertilizers (Figure 4E,F).

### 2.4. Modulation of the Aromatic Profiles in Cherry Tomatoes Achieved Using HNW

**Figure 5:**
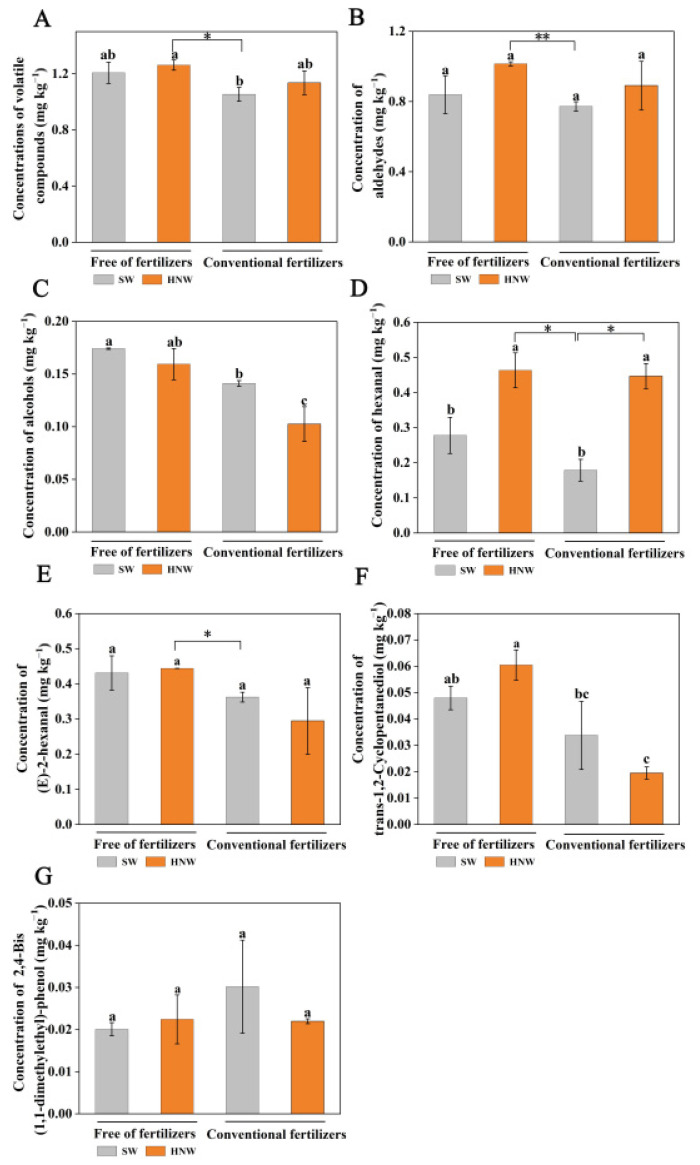
Effects of HNW on the concentrations of volatile compounds (**A**), aldehydes (**B**), alcohols (**C**), hexanal (**D**), E-2-hexenal (**E**), trans-1,2-Cyclopentanediol (**F**), and 2,4-Bis(1,1-dimethylethyl)-phenol (**G**) in cherry tomatoes with/without fertilizers (Shanghai, 2021). Values are mean ± SD of three independent experiments. The asterisks *, ** indicate significant differences at *p* < 0.05 and *p* < 0.01, respectively (*t*-test). The different letters indicate significant differences at *p* < 0.05 (one-way ANOVA; Duncan’s multiple range tests). SW: surface water; HNW: hydrogen nanobubble water.

In this experiment, a total of 49 major volatile compounds was identified, including aldehydes, alcohols, esters, phenols, etc. Among these, the proportion of aldehydes and alcohols was 77.1% and 12.3%, respectively (Supplementary Table S1). With or without fertilizers, HNW irrigation increased the content of total volatile compounds by 8.0% and 4.4%, respectively (Figure 5A). The application of fertilizer alone reduced the content of total volatile compounds (−14.9%) compared with the SW treatment. Accordingly, the treatment of HNW without fertilizers could neutralize the negative effect of fertilizers alone on total volatile compounds (20.0%, *p* < 0.05).

Similarly, HNW irrigation increased the content of aldehydes (by 15.4% and 10.8%, respectively; Figure 5B), hexanal (by 150.2% and 41.5%, respectively, *p* < 0.05; Figure 5D), (E)-2-hexanal (3.0%; Figure 5E), and trans-1,2-cyclopentanediol (26.1%; Figure 5F) both with (except for (E)-2-hexanal and trans-1,2-cyclopentanediol) and without fertilizers. The treatment of HNW without fertilizers showed a greater effect than that of fertilizers alone on aldehydes (*p* < 0.01), including hexanal (*p* < 0.05), (E)-2-hexanal (*p* < 0.05), and trans-1,2-cyclopentanediol (*p* < 0.05), respectively. However, the application of fertilizer alone had a negative effect on the above compounds other than 2,4-bis(1,1-dimethylethyl)-phenol (increased by 50.3%; Figure 5G) compared with the SW treatment. Meanwhile, no significant alteration was observed in the changes of alcohols (Figure 5C), and 2,4-bis(1,1-dimethylethyl)-phenol. We also noticed that in the presence of fertilizers, HNW addition could significantly increase the hexanal level, but decrease the content of alcohols (*p* < 0.05).

### 2.5. The Absorption of Soil Elements was Influenced by HNW

**Figure 6:**
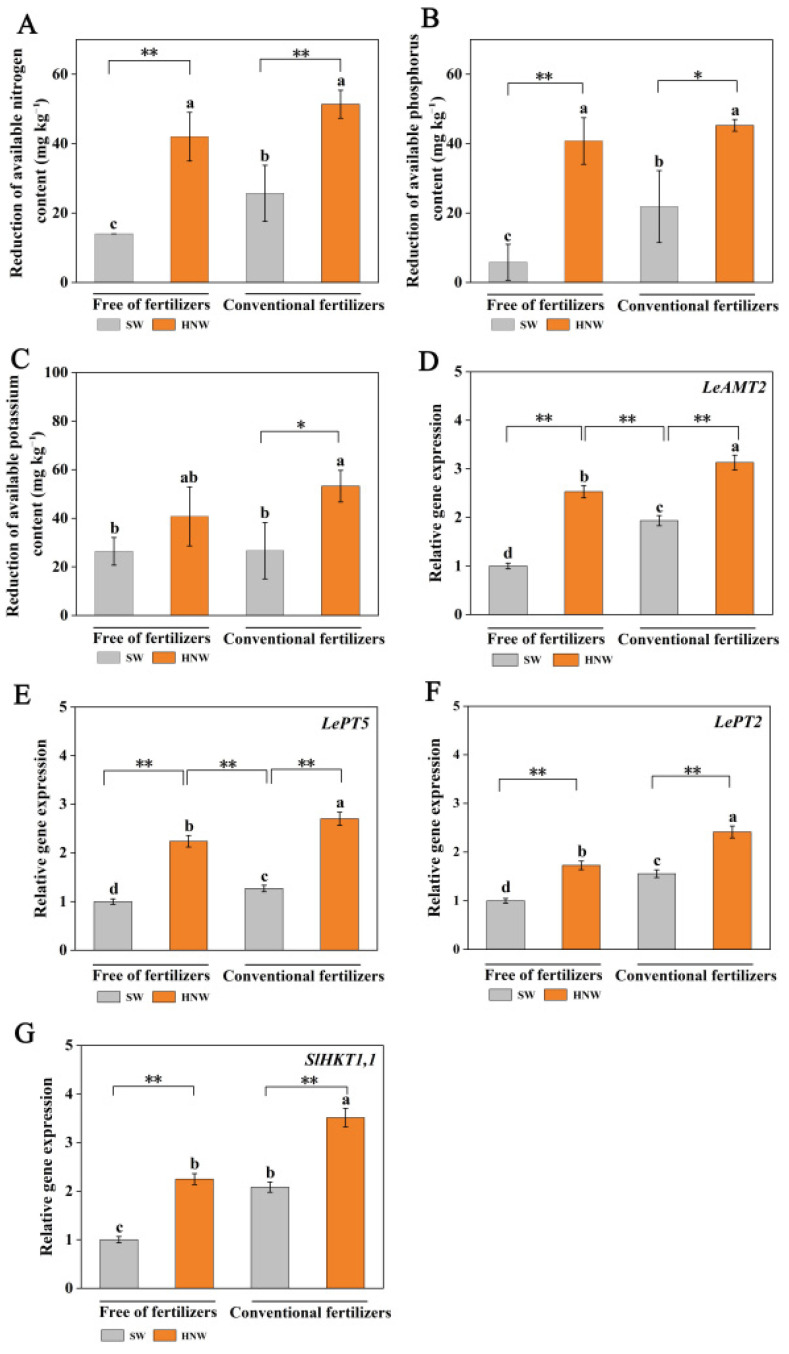
Effects of HNW on the absorption of soil available nitrogen (**A**), phosphorus (**B**), and potassium (**C**) and the expression level of *LeAMT2* (**D**), *LePT5* (**E**), *LePT2* (**F**), and *SlHKT1,1* (**G**) in cherry tomatoes with/without fertilizers (Shanghai, 2021). Values are mean ± SD of three independent experiments. The asterisks *, ** indicate significant differences at *p* < 0.05 and *p* < 0.01, respectively (*t*-test). The different letters indicate significant differences at *p* < 0.05 (one-way ANOVA; Duncan’s multiple range tests). SW: surface water; HNW: hydrogen nanobubble water.

Before planting, the content of soil available nitrogen (N), phosphorus (P), and potassium (K) in different treatments was basically at the same level (Figure S1). Subsequent results showed that without fertilizers, the HNW treatment enhanced the decrease in soi available N, P, and K contents (especially N and P, *p* < 0.01) in comparison with surface water, and the above-mentioned effects in soil available NP consumption achieved using HNW were more pronounced than those treated with fertilizers alone (*p* < 0.05; Figure 6A–C). Meanwhile, in the presence of fertilizers, HNW irrigation exhibited similar effects on the reduction in available N (*p* < 0.01), P (*p* < 0.05), and K (*p* < 0.05) contents.

The expression levels of the genes involved in plant N, P, and K accumulation in plants were further investigated (Figure 6D–G). They included a NH_4_^+^ transporter gene (*LeAMT2*), two phosphate transporters genes (*LePT2* and *LePT5*), and a potassium transporter (*SlHKT1,1*) [28,29]. As expected, the changes in transcript levels of the four genes above were consistent with the reduction in soil available NPK contents. These results indicated that a preharvest HNW application might positively improve NPK absorption in cherry tomatoes.

### 2.6. Principal Component Analysis

**Figure 7:**
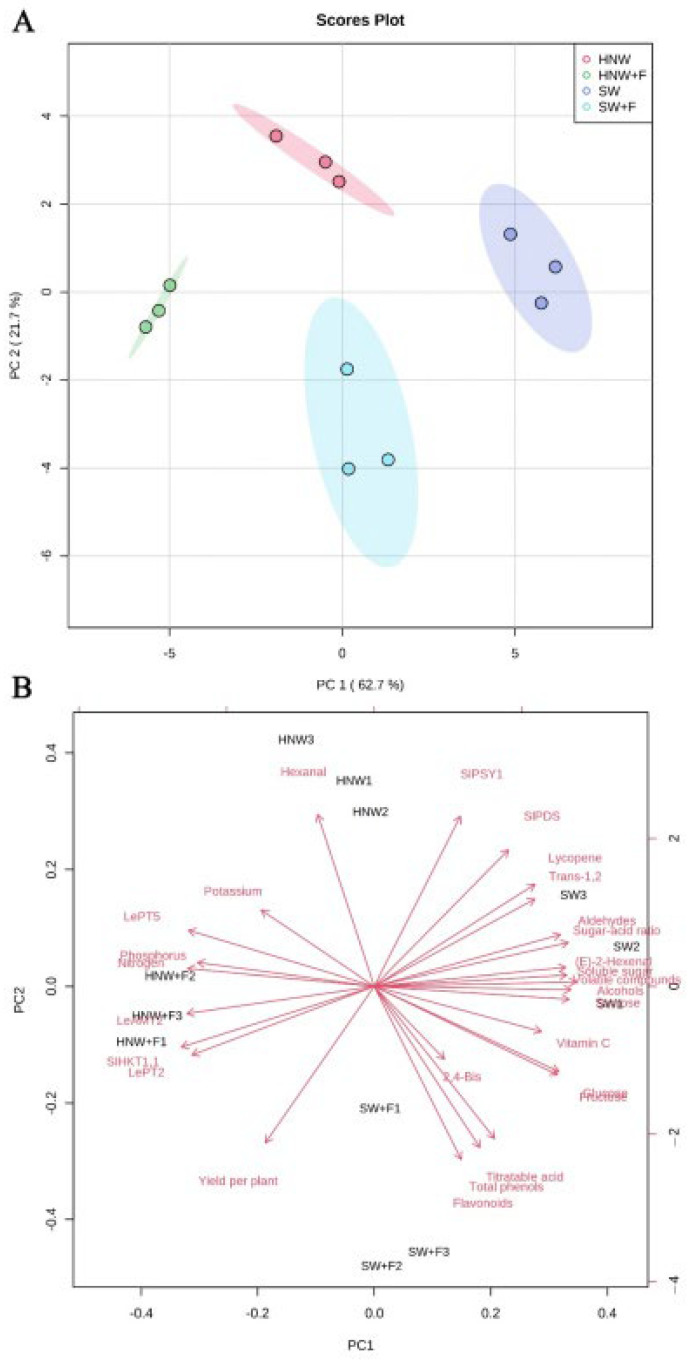
Scores plot (**A**) and biplot (**B**) of principal component analysis (PCA) for effects of HNW on yield and quality in cherry tomatoes with/without fertilizers (Shanghai, 2021). HNW: hydrogen nanobubble water; 2,4-Bis: 2,4-Bis(1,1-dimethylethyl)-phenol; HNW + F: hydrogen nanobubble water plus fertilizers; SW: surface water; SW + F: surface water plus fertilizers; Trans-1,2: trans-1,2-cyclopentanediol.

A principal component analysis (PCA) was performed to distinguish between the above-mentioned four treatments. The first two components explained 84.4% of the total variance (Figure 7A). Meanwhile, the four treatments were clearly separated; the SW and HNW + F treatments were distinguished on PC 1, while PC 2 discriminated the SW + F and HNW treatments, indicating that there were distinct differences. As shown in the biplot (Figure 7B), the quality characters (including sugars, volatiles, titratable acid, total phenols, flavonoids, vitamin C, and lycopene contents, and lycopene biosynthesis-related gene expression) were positively correlated with PC 1, whereas the yield per plant, soil NPK reduction and related gene expression (including *LeAMT2*, *SlHKT1,1*, *LePT2*, and *LePT5*), and hexanal were negatively correlated. Moreover, there were further positive correlations among the quality characters. Similarly, results for the yield showed positive correlations with the NP absorption and expression of *LeAMT2*, *SlHKT1,1*, and *LePT2.* However, the yield was negatively correlated with soluble sugars, the sugar–acid ratio, and volatiles (including alcohols, aldehydes, (E)-2-hexanal, and hexanal) and lycopene contents.

## 3. Discussion

As an environmentally benign gas, H_2_ plays a major role in promoting plant growth, and improving crop yield and nutritional quality [30]. Both H_2_ gas-treated soil and conventional HRW irrigation have been proposed and observed to improve the yield of crops [12,14]. However, H_2_ applied in gas form is not practical in the field (e.g., due to its flammability), and H_2_ is effective in HRW for less than 6 h [11]. Under our trial conditions, the residence time of H_2_ as HNW was approximately 12 h, which was consistent with a previous study [19], and twice as long as that in HRW (Figure S2).

Although the mechanism of H_2_ fertilization using its gas in enhancing the plant yield has yet to be fully understood, it can most probably be attributed to the enhanced growth of H_2_-oxidizing bacteria in the soil. These microorganisms may improve the nutrient status of soil, and enhance the plants growth regulator balance or disease resistance [12,31]. It was previously reported that H_2_ exposure can increase soil carbon deposition [32] and the synthesis of soil enzymes such as catalase, dehydrogenase, and urease [33]. These results reflected the possibility that H_2_ improves soil fertility by inducing the metabolic activity of beneficial bacteria. Therefore, we chose the four greenhouses closely (Figure S3) and with the same crop rotation (tomato) to avoid differences in climatic, illumination, and microorganism conditions. Importantly, we tested the nutrition of soil samples, and the results (Figure S1) showed that the initial soil conditions of the four greenhouses were similar in terms of the key nutrition, including the available nitrogen, phosphorus, and potassium.

Two-year field trials clearly showed that HNW improved the yield of greenhouse cherry tomatoes and that this was more pronounced than when cultured with fertilizers (Figure 1 and Figure 2). Moreover, an additional effect on the cherry tomato yield was observed in the presence of HNW plus fertilizers.

NPK are the principal nutrients typically supplied to plants, so the absorption and utilization efficiency of these elements controls the crop yield [34]. It has been reported that HNW increased the transcription of the genes related to the absorption of NPK in rice, including *NRT2.3*, *NiR*, *ARE1*, *NLP4*, and *AKT1* transcripts [21]. In this study, it was clearly observed that NPK transport-related genes in plants (especially *LeAMT2*, *LePT2*, and *SlHKT1,1*) were positively correlated with soil NPK reduction and the yield of tomato fruits (Figure 7B).

It has been reported that the combined application of a microbial consortium and fertilizer increased the soil available NPK content, and promoted NPK absorption by sugarcane plants, thereby promoting plant growth and increasing sugarcane and sugar yields [35]. A previous study showed that a high level of H_2_-oxidizing bacteria in H_2_-treated soil increased the plant biomass and promoted plant growth [31]. Since HNW in this study was irrigated at the early growth stage of cherry tomatoes, we deduced that the beneficial roles of HNW in improving soil NPK absorption and the cherry tomato yield may be partially associated with H_2_ impacting soil microbes. This hypothesis requires further investigation on the interaction of plants and microbes in response to H_2_. Since in our experimental conditions HNW may differentially increase the expression levels of the four genes and soil available NPK consumption (Figure 6), we further proposed that these changes may also be partially responsible for the promotion of the cherry tomato yield in the absence/presence of fertilizers (Figure 1 and Figure 2).

Sugars, acids, and their ratio are major contributors to fruit taste [23,36]. A high sugar–acid ratio enhances the desirable sweet perception. A previous study showed that HNW increased the sugar–acid ratio as a result of the increased sugar content and decreased titratable acid content in strawberries [22]. In this study, without fertilizer addition, HNW increased the sugar–acid ratio by increasing the total soluble sugar content (especially of fructose; Figure 3A,D). A previous study pointed out that nitrogen fertilization affected the activities of enzymes directly related to acid metabolism in fruit, thereby changing the acid content [37]. Consistently, the application of fertilizers increased titratable acid content, thus decreasing the sugar–acid ratio (Figure 3B,C). However, HNW abolished the above negative effect of fertilizers by regulating the balance of sugar and acid (Figure 3A–C), which was consistent with a previous study in strawberry plants [22].

Lycopene, vitamin C, and total phenols and flavonoids are important antioxidants in fruits and vegetables [25]. A previous study observed that HRW enhanced the tolerance against UV-B stress, which was associated with the improvement in flavonoids profiles in alfalfa seedlings [38]. In addition, HRW can also alleviate oxidative damage by increasing the content of vitamin C, total phenols, and flavonoids, resulting in the prolonged shelf life of tomatoes [39], daylily buds [14], and lychee [40]. Consistently, our results showed that HNW alone increased the lycopene content in tomato fruits (Figure 4A). This is a new finding. HNW’s control of the lycopene increase was further supported by the up-regulation of *SlPSY1* and *SlPDS* transcripts (Figure 4E,F), two lycopene synthesis genes [41], and the results of the PCA (Figure 7B). Therefore, it was suggested that the two genes mentioned above might be the target genes responsible for HNW-triggered lycopene accumulation.

Although more than 400 volatile compounds have been identified in tomato fruit, current studies show that the most important compounds of aldehydes, such as hexanal and (E)-2-hexenal; alcohols, such as trans-1,2-cyclopentanediol; and phenols, such as 2,4-bis (1,1-dimethylethyl)-phenol, play key roles in the tomato aroma [42]. Aldehydes are the most dominant, by giving off the ‘fresh green’ odor [43]. A recent study showed that the contents of total volatile compounds, and aldehydes, such as hexanal and (E)-2-hexenal, were increased using a preharvest HNW application in strawberries [22]. In this report, we discovered that HNW also increased the hexanal content in cherry tomatoes with/without fertilizers (Figure 5D), reflecting the possible common mechanism.

Furthermore, a positive correlation between soluble sugars and volatile compounds contents in cherry tomatoes (Figure 7B) was consistent with the previous studies on strawberries [22,44]. Since the important volatile compounds, such as esters, furanones, and terpenes, are present in the form of glycosides in cells, and the precursors of those were sugars [45,46], aromatic volatiles in tomato fruits may be positively associated with sugars. In addition, sugars and volatile compounds are known to be the important factors influencing sweetness perception [47,48]. Consumer liking was associated with sweetness and aroma intensity [44]. Therefore, HNW-increased sugars and volatile compounds contents in cherry tomatoes should be more attractive for consumers.

Under the condition of limited fruit carbohydrates, plants preferentially utilize the carbohydrates transported into the fruits to form carbon skeletons, which may lead to a lower fruit quality but a higher yield [49]. Consistently, in this study, a negative correlation was observed between the yield and quality characters, including soluble sugars, volatile compounds, and lycopene contents, etc. (Figure 7B). As expected, compound fertilizers promoted the yield of cherry tomatoes, but undesirably reduced fruit sugars, lycopene, and volatile contents [50].

**Figure 8:**
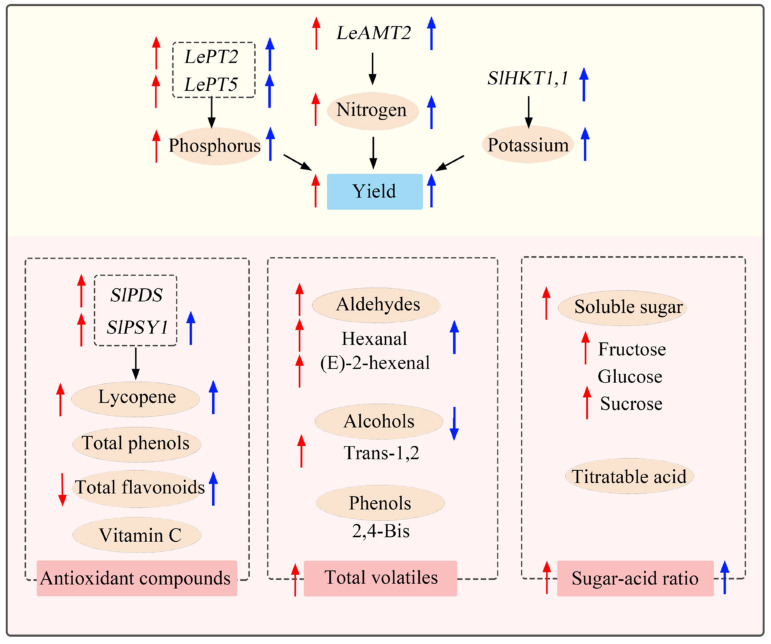
Proposed schematic model of HNW-improved yield and quality in cherry tomatoes. Compared with surface water plus fertilizers, red arrows (left) indicate the effects achieved using HNW without fertilizers, and blue arrows (right) indicate the effects achieved using HNW plus fertilizers. AMT2: ammonium transporter 2; 2,4-Bis: 2,4-Bis(1,1-dimethylethyl)-phenol; HKT1,1: high-affinity K^+^ channel transporter; PT2/5: phosphate transporter 2/5; PDS: phytoene desaturase; PSY: phytoene synthase; Trans-1,2: trans-1,2-cyclopentanediol.

Together, as shown in the schematic model summarizing the effects of yield and quality in response to HNW (Figure 8), it is worth noting that hydrogen fertilization with HNW may not only improve cherry tomato yield, which was better than grown with fertilizers alone to some extent, but also partly weaken the negative fertilizer effects on the sugar–acid ratio, volatile compounds, and lycopene contents. The above effects of HNW may be attributable to the regulation of plant NPK absorption, carbohydrate, and secondary metabolism.

## 4. Materials and Methods

### 4.1. Plant Materials and Experimental Design

The field experiment was carried out at the Qingpu Agriculture Base, Shanghai, China (longitude 121°01′ E and latitude 31°02′ N; 2021) and White Horse Agriculture Base, Lishui District, Nanjing, Jiangsu, China (longitude 119°17′ E and latitude 31°58′ N; 2023). The cherry tomato ‘Jintong’ (*Lycopersicon esculentum* var. *cerasiforme* ‘Jintong’) was planted on 21 January 2021 and 7 March 2023, respectively. Considering the fugitiveness of H_2_, four greenhouses were used for the experiment, and three plots (each 20 m long and 3 m wide) were randomly selected as replicates for each treatment per greenhouse (80 m long and 6 m wide). Before fertilizing, contents of soil available nitrogen (N), phosphorus (P), and potassium (K) in four greenhouses were basically at the same level (Shanghai, 2021): available nitrogen content 215.8 ± 4.5 mg kg^−1^; available phosphorus content 410.7 ± 3.7 mg kg^−1^; available potassium content 406.4 ± 3.8 mg kg^−1^ (Figure S1). Furthermore, the number of cherry tomato plants in one treatment per greenhouse was 1288.

Four treatments were arranged, including (1) irrigation with surface water (SW) and free of fertilization; (2) irrigation with SW and normal fertilization; (3) irrigation with HNW and free of fertilization; and (4) irrigation with HNW and normal fertilization.

Fertilizers were applied conventionally in the required treatments. A compound fertilizer (Nitrophoska^®^, Stellenbosch, South Africa, 15-15-15, 15% N, 15% P_2_O_5_, 15% K_2_O) was used as the base fertilizer on 10 December 2020 (Shanghai) and 7 January 2023 (Nanjing) without topdressing. The amount of fertilizers applied was 50 kg (15.6 g m^−2^ for N/P_2_O_5_/ K_2_O) per greenhouse (80 m long and 6 m wide). Furthermore, no pesticides were applied during the plant growth stages. Flood irrigation with HNW was carried out once on the day after planting at 10 t h^−1^ flow rate for 0.5 h per greenhouse for HNW treatment. Meanwhile, irrigation with the same amount of SW was set as the control. Subsequent field operations were in accordance with the conventional agricultural managements.

### 4.2. Preparation of HNW

In this experiment, HNW (with ~300 nm nanobubbles) was prepared with a H_2_ nanobubbles generator (Air Liquide (China) R&D Co., Ltd., Shanghai, China). The dissolved H_2_ concentration was measured using an ENH-2000 portable dissolved H_2_ meter (TRUSTLEX, Osaka, Japan) that was calibrated with gas chromatography. The fresh HNW contained 1.0 mg L^−1^ H_2_, and it remained in HNW for ~12 h (Figure S2).

### 4.3. Determination of Cherry Tomato Yield

The cherry tomato fruits were harvested once a week from 16 April to 15 June 2021 and from 1 June to 25 July 2023, respectively, when their color changed from green to yellow. Afterwards, the yield per plant was calculated.

The freshly picked tomatoes were placed in sampling bags during the fruiting stage and transported back to the laboratory in the dark at room temperature. The yield parameters were analyzed in triplicate, and at least 10 plants/treatment/repeat were used (except Figure 2D). Among these, we measured the single fruit weight also in triplicate, calculating the average of 30 tomato fruits/treatment/repeat. For each treatment, ten or twenty fruits from different plants were mixed and ground using a mill (A11, IKA, Staufen, Germany), and stored at −80 °C. Pooled samples were split into three replicates for the biochemical and molecular analysis.

### 4.4. Evaluation of Fructose, Glucose, Sucrose, Titratable Acid, and Soluble Sugar Contents

Following a previous method [51], twenty fruits from four greenhouses with different treatments were ground using a mill (A11, IKA, Staufen, Germany) and stored at −80 °C, and pooled samples were split into three replicates for the following analysis. Fructose and glucose contents were detected using a High Performance Liquid Chromatograph (HPLC; Infinity 1260; Agilent, Santa Clara, CA, USA) with a 250 mm × 4.6 mm ZORBAX column (Agilent, USA) at 40 °C. Elution was used with 75% acetonitrile (*v*/*v*), with 20 μL injection and 1.3 mL min^−1^ for the flow rate. The amount of sucrose was assayed according to the Lane–Eynon method [52]. The test solution was titrated with Fehling’s solution, containing a methylene blue indicator.

The measurement of titratable acid was achieved by the sample being titrated with 0.1 M NaOH to an end-point pH of 8.2 [53]. The results were expressed as g kg^−1^ fresh weight.

The content of soluble sugars was determined with anthrone–sulfuric acid colorimetric method [54]. Firstly, the samples were mixed with 2% (*w*/*v*) anthrone regent/concentrated sulfuric acid (0.5:5, *v*/*v*) solution, and then incubated in boiling water for 10 min. After centrifugation, the absorbance of the supernatant was recorded at 620 nm. The soluble sugar content was expressed as g kg^−1^ fresh weight calculated using the standard curve of sucrose.

### 4.5. Determination of Lycopene, Vitamin C, Total Phenols, and Flavonoids Contents

Lycopene, vitamin C, total phenols, and flavonoids were averaged from three independent samples prepared by pooling 20 individual fruits from different plants for each treatment. Lycopene was exacted by using mixed solvent extraction method described previously [55]. The fruit samples (1.0 g) added with the solution of acetone/petroleum (5:5, *v*/*v*) were incubated in a 30 °C water bath for 15 min. Afterwards, the absorbance was determined at 472 nm. The standard curve was obtained by adding different concentrations of a lycopene standard solution.

Vitamin C content was detected with the HPLC system (D-2000, Hitachi, Ltd., Tokyo, Japan). According to the previous method [56], the sample homogenate was extracted with oxalic acid solution under dark conditions for 10 min. After centrifugation and filtration with a 0.45 μm water filtration membrane, the test solution was prepared for detection. Vitamin C content expressed as g kg^−1^ fresh weight was calculated from the standard curve of vitamin C.

The content of total phenols was estimated using the Folin–Ciocalteu reagent and the absorbance was measured at 765 nm [57]. The results were obtained from a standard curve for gallic acid, expressed as g kg^−1^ fresh weight.

The flavonoids content was determined using the aluminum chloride colorimetric method [58,59]. The samples (0.1 g) were dispersed in 1 mL of deionized water, and then mixed with a solution of 95% alcohol, 10% aluminum chloride hexahydrate, 1 M potassium acetate, and deionized water (1.5:0.1:0.1:2.8, *v*/*v*/*v*/*v*).

### 4.6. Extraction and Analyses of Aromatic Compounds

Headspace solid phase microextraction was used to sample volatiles of tomatoes according to a previous method [22]. Ten fruits were ground together using a mill (A11, IKA, Germany). These samples were split into three aliquots and all replicates of 5 g were processed using following methods. The samples were added with 1 g of sodium chloride, containing 10 μL of 2-nonanone (internal standard; dilute 5 × 10^3^ times; Macklin, China) in a 20 mL vial. Volatiles were collected using a 1cm DVB/CAR/PDMS Stable Flex fiber (50/30 μm; Supelco, PA, USA) in a 50 °C water bath for 30 min after a 50 °C equilibration for 15 min. Afterwards, the analysis was performed on a 320-MS gas chromatography-mass spectrometer (GC-MS, Bruker, Germany) with a BR-5 ms column (30 m × 0.25 mm ID × 0.25 μm). The program of GC-MS was set according to a previous method [22]. Chromatographic profiles of volatiles were identified using spectral library comparison (NIST standard library). The relative content of the compound referred to the internal standard and expressed as mg kg^−1^ fresh weight.

### 4.7. Determination of Soil Available Nitrogen (N), Potassium (K), and Phosphorus (P) Contents

Soil samples were collected on 4 January 2021 (before planting) and 8 June 2021 (later stage of fruiting) using the five-point sampling method. The soils were collected at 5–10 cm depth and mixed from 5 points in each treatment. Subsequently, the air-dried soil samples were filtered through a 2 mm sieve and divided into three replicates for the following analysis.

Available N content was measured according to the previous methods [60]. The soil samples were hydrolyzed with ferrous sulfate under alkaline conditions and then the hydrolyzed and nitrate N were converted to ammonium nitrogen. The diffusion ammonium nitrogen was absorbed by boric acid solution and titrated with standard acid to obtain the content of soil available nitrogen.

Available K content was estimated using Inductively Coupled Plasma Optical Emission Spectrometry (ICP-OES; Optima 8000, Perkin Elmer, Waltham, MA, USA) mentioned previously [61]. The soil sample (5 g) was added with 50 mL of ammonium acetate solution (1 mol/L, pH 7.0). After filtering, the test solution was detected using ICP-OES.

Available P was extracted with the solution of ammonium fluoride–hydrochloric acid and incubated at 25 °C with shaking for 30 min, and finally estimated using ICP-OES. The amounts of P and K were calculated from standard curves of P and K standard solution, respectively.

### 4.8. Real-Time Fluorescence Quantitative PCR Analysis

Total RNA was extracted from the fruits and roots of the plants during the fruiting stage (4 May 2021). Root samples were immediately frozen in liquid nitrogen after they were taken. The samples were mixed and ground using a mill, and stored at −80 °C. Pooled samples were split into three replicates for the following analysis. The exaction of total RNA was performed using a TransZol Plant kit (TransGen Biotech, Beijing, China). Afterwards, the concentration and purity of RNA were measured using a NanoDrop 2000 Spectrophotometer (Thermo Fisher Scientific, Waltham, MA, USA). cDNA was synthesized based on the manufacturer’s instructions of HiScript III-RT SuperMix kit (+gDNA wiper) for qPCR (Vazyme, Nanjing, China). The TransStart^®^ Top Green qPCR SuperMix kit from TransGen was used for qPCR, which was conducted on a Mastercycler^®^ EP Realplex Real-time PCR system from Eppendorf. The *18S rRNA* and *Actin* (*SlACT*) were used as reference genes. The primer sequences are shown in Supplementary Table S2. Relative gene expression levels were determined with the 2^−∆∆CT^ method [62].

### 4.9. Statistical Analysis

The results were analyzed using SPSS 24.0 software to express results a mean ± standard deviation (SD) for three independent experiments. One-way analysis of variance (ANOVA), Duncan’s multiple-range test, and *t*-test were used for data analysis. Differences were considered significant at * *p* < 0.05 and ** *p* < 0.01.

The data were normalized and scaled (sum-based normalization, square-root transformation, and auto-scaling procedures). Principal component analysis (PCA) was carried out using MetaboAnalyst 5.0 (https://www.metaboanalyst.ca, accessed on 30 June 2022).

## 5. Conclusions

The sustained improvement of horticultural yields requires NPK fertilizers, which could be offset using healthier and cleaner alternatives to maintain fruit consumer quality. Compared to solid H_2_ storage materials such as MgH_2_, AB, and other nanoparticles, HNW will not bring additional elements. Recently, genetic and molecular evidence showed that molecular hydrogen not only influenced root organogenesis [63,64], but also increased nitrogen use efficiency (NUE) by targeting nitrate reductase [65]. Consistently, in this report, we revealed that the preharvest application of hydrogen fertilization with HNW exhibited a fertilization effect on the cherry tomato yield and improved its quality, even to some extent as an alternative for the conventional fertilizers. Moreover, the production costs of renewable H_2_ are reducing to USD 0.7–1.6/kg H_2_ before 2050 on a global scale [66]. Consequently, HNW may provide an easy, affordable, and environment friendly solution for the reduction in fertilizer use, thus improving agricultural sustainability.

## Data Availability

Data are contained within the article and Supplementary Materials.

## Supplementary Materials

The following supporting information can be downloaded at: https://www.mdpi.com/article/10.3390/plants13030443/s1, Table S1: Profiles of volatiles in cherry tomatoes (2021); Table S2: Primers of qPCR used in this study. Figure S1: The contents of available nitrogen, available phosphorus, and available potassium in soil of four greenhouses before fertilizing (Shanghai, 2021); Figure S2: Changes in H_2_ content of fresh HRW and HNW; Figure S3: The design and location information of the four greenhouses (Shanghai, 2021).
