# Supplementary material for: Hydrogen Fertilization with Hydrogen Nanobubble Water Improves Yield and Quality of Cherry Tomatoes Compared to the Conventional Fertilizers
Source: Plants (Basel). 2024 Feb 2;13(3):443. doi: 10.3390/plants13030443 (PMC10857181; doi:10.3390/plants13030443)
Supplement: Supplementary file 1 [file plants-13-00443-s001.zip › plants-2791469-supplementary.pdf]

Supplemental Materials

Table S1. Profiles of volatiles in cherry tomatoes (2021). Values are mean ± SD of three independent experiments. ND: not detected; SW: surface water; HNW: hydrogen nanobubble water; SW + F: surface water plus fertilizers; HNW+F: hydrogen nanobubble water plus fertilizers.

Code	Family	Compound	Concentration (mg kg^{-1})			
			SW	HNW	SW+F	HNW+F
1	Aldehydes	Hexanal	0.278±0.052	0.463±0.050	0.179±0.031	0.447±0.036
2	(average proportion: 77.1% ±1.6%)	(E)-2-Hexenal	0.431±0.049	0.444±0.000	0.362±0.014	0.294±0.094
3		2-Methyl-4-pentenal	0.122±0.009	0.031±0.044	0.121±0.007	0.017±0.024
4		Methional	0.002±0.003	0.004±0.006	0.007±0.001	0.003±0.005
5		Benzeneacetaldehyde	0.035±0.001	0.008±0.011	0.043±0.001	0.034±0.008
6		Decanal	0.004±0.001	0.002±0.003	0.005±0.001	0.006±0.000
7		(E,E)-2,4-Hexadienal	0.012±0.001	0.022±0.019	0.015±0.002	0.006±0.001
8		(Z)-2-Heptenal	0.007±0.001	0.002±0.003	0.003±0.004	0.016±0.007
9		(E)-4-Oxohex-2-enal	0.022±0.007	0.024±0.012	0.034±0.001	0.012±0.016
10		Nonanal, 3-(methylthio)-	0.002±0.003	ND	ND	0.002±0.003
11		Nonanal	ND	0.014±0.019	ND	0.047±0.029
12		5-Methyl-hexanal	ND	ND	0.002±0.003	ND
13		(E)-2-Octenal	ND	ND	ND	0.008±0.011
14		(E)-2-Nonenal	0.002±0.003	ND	ND	ND
15	Alcohols	trans-1,2-Cyclopentanediol	0.048±0.004	0.060±0.006	0.034±0.013	0.020±0.002
16	(average proportion: 12.3% ± 4.8%)	2-Ethyl-1-hexanol	0.010±0.000	0.004±0.006	0.007±0.001	0.004±0.006
17		Phenylethyl alcohol	0.075±0.002	0.075±0.001	0.089±0.005	0.069±0.011

18		2,4,6-Trimethyl-1,6-heptadien-4-ol	0.023±0.000	0.009±0.012	0.006±0.009	0.007±0.010
19		Benzene-1,2-diol, 4-(2-guanidinothiazol-4-yl)-	0.005±0.000	0.003±0.004	0.004±0.000	ND
20		3-Hepten-1-ol	0.001±0.002	0.008±0.004	ND	ND
21		cis-1,3-Cyclopentanediol	0.004±0.006	ND	ND	ND
22		cis-2-Methylcyclohexanol	0.004±0.001	ND	ND	ND
23		5-Methyl-5-hexen-2-ol	0.002±0.003	ND	ND	ND
24		E,E-2,6-Dimethyl-3,5,7-octatriene-2-ol	0.001±0.002	ND	ND	0.003±0.001
25	Esters	Carbamic acid, (2-methylphenyl)methyl ester	0.019±0.003	0.009±0.002	0.020±0.002	0.026±0.005
26	(average proportion: 1.9% ± 1.5%)	Decanoic acid, ethyl ester	0.004±0.000	ND	0.002±0.004	0.005±0.000
27		Isobutyl acrylate	ND	0.002±0.003	ND	0.001±0.002
28		Oxalic acid, isohexyl neopentyl ester	ND	ND	ND	0.001±0.001
29	Phenols (average proportion: 2.1% ± 1.2%)	2,4-Bis(1,1-dimethylethyl)-phenol	0.020±0.002	0.022±0.006	0.030±0.011	0.022±0.001
30	Others (average proportion: 6.6% ± 2.9%)	L-Isoleucine	0.012±0.016	0.042±0.031	ND	ND
31		1,6-Anhydro-3,4-dideoxy-.beta.-D-manno-hexapyranose	0.013±0.018	ND	ND	0.012±0.006
32		Benzene, 1-(1-hydroxyheptyl)-3-[1-(tetrahydropyran-2-yloxy)heptyl]-	ND	0.006±0.008	0.005±0.007	ND
33		Oxetane, 2-methyl-4-propyl-	0.006±0.000	0.003±0.004	0.002±0.003	0.002±0.003
34		Benzyl nitrile	0.014±0.000	0.004±0.005	0.011±0.000	0.022±0.011
35		Octyl-oxirane	0.005±0.000	ND	ND	0.004±0.005
36		Propanoic acid, 3-hydroxy-, hydrazide	ND	ND	0.009±0.012	0.002±0.003
37		4-(2,2,6-Trimethyl-7-oxabicyclo[4.1.0]hept-1-yl)-3-buten-2-one	0.003±0.004	ND	ND	0.003±0.001
38		Bis(3-methylbutyl) fluorene-2,7-disulfonate	0.004±0.000	ND	0.004±0.000	0.004±0.005

39	Hydroxylamine, O-decyl-	0.006±0.001	ND	0.005±0.001	0.003±0.004
40	Decane, 6-ethyl-2-methyl-	ND	ND	ND	0.001±0.001
41	trans-β-Ionone	ND	ND	ND	0.003±0.000
42	1H-Pyrrole, 2,5-dihydro-1-nitroso-	0.007±0.010	ND	0.036±0.011	ND
43	2-Isobutylthiazole	ND	ND	ND	0.022±0.022
44	Thieno[2,3-c]furan-3-carbonitrile, 2-amino-4,6-dihydro-4,4,6,6-tetramethyl-	ND	ND	0.003±0.005	ND
45	1,2-Epoxyundecane	ND	ND	0.008±0.000	ND
46	Valeric acid hydrazide	ND	ND	0.004±0.000	ND
47	Methyl 2-methoxypropenoate	0.005±0.007	ND	ND	ND
48	4-Hydroxybutyric acid hydrazide	ND	ND	0.001±0.002	ND
49	Hydrazinecarboxamide	ND	ND	ND	0.007±0.010

Table S2. Primers of qPCR used in this study.

Gene	Forward primer (5’–3’)	Reverse primer (5’–3’)
SlACT (NM_001330119.1)	CGGTGACCACTTTCCGATCT	TCCTCACCGTCAGCCATTTT
18S rRNA (XM_004241429.4)	GGGCATTCGTATTTCATAGTCAGA	GTTCTTGATTAATGAAAACATCCT
SlPSY1 (NM_001347838.1)	TGGCCCAAACGCATCATATA	CACCATCGAGCATGTCAAATG
SlPDS (NM_001247166.2)	TGCCAAACCACCACAAATTTCA	CCAATTTGAGGCATTTTACTCGGA
LeAMT2 (NM_001247324.2)	TTGTACCGCCGCTCTGACAACTTT	CATGGCTCAACAACTGCACAACCT
LePT2 (NM_001247114.1)	CATTGGACACTGGAGGCTAACC	ATAAGAACCCATACGCTCCCA
LePT5 (XM_004240903.4)	GGCGAATGAAGATGCCTGAAAC	TACCAATTAAGTGATGTCCGTG
SlHKT1,1(NM_001308344.1)	TGTTTTGTGCTTTGGAGTGGA	GGGGGTGAAAGAGTGGAGAT

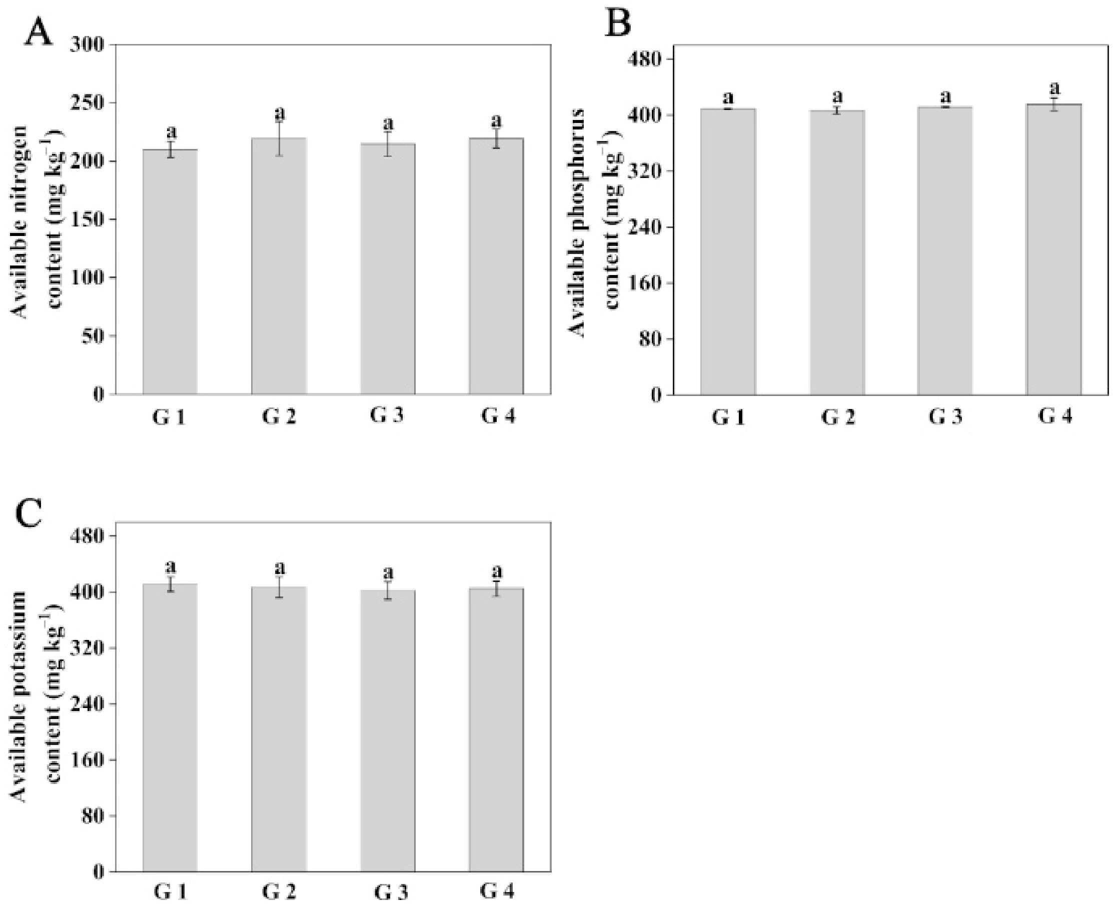

Figure S1. The contents of available nitrogen (A), available phosphorus (B), and available potassium (C) in soil of four greenhouses before fertilizing (Shanghai, 2021). The different letters indicate significant differences at $p < 0.05$ (one–way ANOVA; Duncan's multiple range tests). G 1: greenhouse 1; G 2: greenhouse 2; G 3: greenhouse 3; G 4: greenhouse 4.

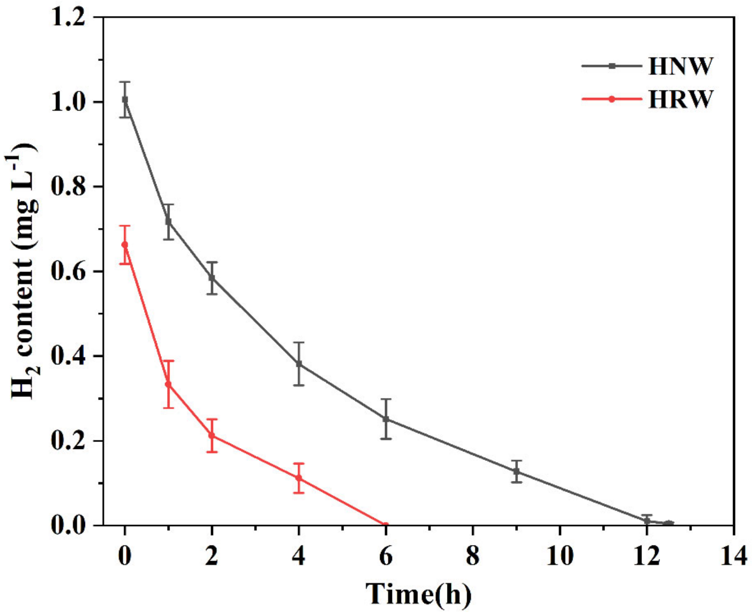

Figure S2. Changes in H_2 content of fresh HRW and HNW. HRW: hydrogen-rich water; HNW: hydrogen nanobubble water.

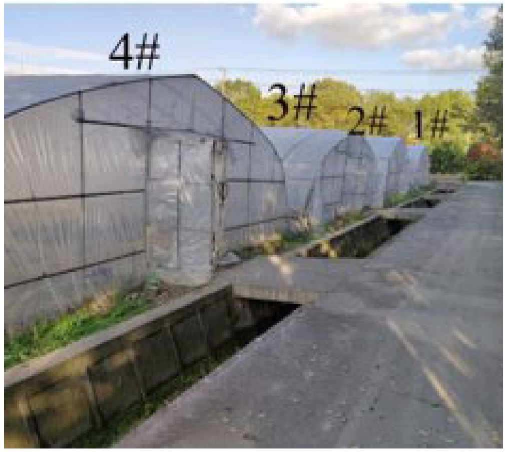

Figure S3. The design and location information of the four greenhouses (Shanghai, 2021). 1#: Irrigation with surface water (SW) and normal fertilization; 2#: Irrigation with SW and free of fertilization; 3#: Irrigation with hydrogen nanobubble water (HNW) and normal fertilization; 4#: Irrigation with HNW and free of fertilization.
